# Building capacity for clinical practice guideline development: Experiences from Malawi, Nigeria and South Africa

**DOI:** 10.4102/phcfm.v18i1.5159

**Published:** 2026-07-16

**Authors:** Idriss I. Kallon, Elodie A.S. Besnier, Tamara Kredo, Sara Cooper, Willem Odendaal, Carmen Späth, Emmanuel E. Effa, Ekpereonne B. Esu, Dachi Arikpo, Simon Lewin, Nyanyiwe M. Mbeye, Retsedisitsoe P. Mazibuko, Denny Mabetha, Roselyn Chipojola, Ameer Hohlfeld, Michael McCaul, Anke C. Rohwer

**Affiliations:** 1 Centre for Evidence-based Health Care, Division of Epidemiology and Biostatistics, Faculty of Medicine and Health Sciences, Stellenbosch University, Cape Town, South Africa; 2 Department of Health Sciences in Ålesund, Faculty of Medicine and Health Sciences, Norwegian University of Science and Technology, Ålesund, Norway; 3 Health Systems Research Unit, South African Medical Research Council, Cape Town, South Africa; 4 School of Public Health and Family Medicine, University of Cape Town, Cape Town, South Africa; 5 Department of Medicine, Division of Clinical Pharmacology, Stellenbosch University, Cape Town, South Africa; 6 Division of Epidemiology and Biostatistics, Faculty of Medicine and Health Sciences, Stellenbosch University, Cape Town, South Africa; 7 Cochrane South Africa, South African Medical Research Council, Cape Town, South Africa; 8 Department of Global Health, Faculty of Medicine and Health Sciences, Stellenbosch University, Cape Town, South Africa; 9 Department of Psychiatry, Faculty of Medicine and Health Sciences, Stellenbosch University, Cape Town, South Africa; 10 Department of Internal Medicine, Faculty of Clinical Sciences, University of Calabar, Calabar, Nigeria; 11 Cochrane Nigeria, Calabar Institute of Tropical Diseases Research and Prevention, University of Calabar Teaching Hospital, Calabar, Nigeria; 12 Centre for Epidemic Interventions Research, Norwegian Institute of Public Health, Oslo, Norway; 13 Department of Community and Environmental Health, Evidence Informed Decision-making Centre, School of Global and Public Health, Kamuzu University of Health Sciences, Blantyre, Malawi; 14 South African Medical Research Council, Wits Rural Public Health and Health Transitions Research Unit, School of Public Health, University of the Witwatersrand, Johannesburg, South Africa

**Keywords:** capacity development, clinical practice guideline, guideline development, evidence-to-decision framework, mixed-methods, qualitative, quantitative

## Abstract

**Background:**

Within the Global Evidence, Local Adaptation (GELA) project, we offered training to guideline development group (GDG) and steering group (SG) members in Malawi, Nigeria and South Africa, to build capacity in clinical practice guideline (CPG) development.

**Aim:**

This study aimed to evaluate the capacity development of GDG and SG committee members within GELA.

**Setting:**

Guideline development group members were mainly based at health or academic institutions, while SG members were mostly linked to the health ministries in Malawi, Nigeria and South Africa.

**Methods:**

We used mixed-methods for our summative evaluation, comprising a document analysis, online surveys, semi-structured, in-depth interviews and a structured reflection. For the survey, respondents rated their confidence in knowledge, skills and behaviour before and after GELA. Data were analysed with R studio. We interviewed selected participants from all countries and analysed qualitative data through framework analysis. The author team met in-person to reflect on activities, survey findings and interview data.

**Results:**

Confidence in CPG development skills and related behaviour increased during GELA. Qualitative themes showed an enhanced understanding and valuing of a rigorous guideline development process that should be sustained and institutionalised. The intensive nature of training, limited internet connectivity, time constraints and unmet expectations, were some reported challenges. Participants appreciated increased collaboration and opportunities for future networking. Reflections highlighted the importance of learning by doing.

**Conclusion:**

Global Evidence, Local Adaptation built capacity of GDG and SG members to participate in rigorous CPG development through a multifaceted approach. Continued networking and collaboration of various stakeholders is key to sustain learning.

**Contribution:**

Capacity for rigorous CPG development can be built through a strategic multifaceted capacity development programme and learning by doing.

## Introduction

Clinical practice guidelines (CPGs) have been defined as:

[*S*]tatements that include recommendations intended to optimise patient care. They are informed by a systematic review of evidence and an assessment of the benefits and harms of alternative care options.^1^ p. 25

In addition to considering benefits and harms, other factors such as costs, acceptability, feasibility and equity implications of alternative care options need to be assessed.

Globally, the value of CPGs is increasingly being recognised. This has, firstly, led to a rise in the number of CPGs being produced.^2^ For example, the World Health Organization (WHO) has developed CPGs for all major infectious conditions and for more and more non-communicable conditions. Secondly, the evidence base on CPG methods and processes is growing, with published guidance on CPG development, adaptation, contextualisation, implementation and evaluation. The WHO, the GRADE working group (https://www.gradeworkinggroup.org/) and the Guidelines International Network (https://g-i-n.net/home) are recognised as knowledge leaders in this field and have set international standards for developing guidelines from scratch (‘de novo’ guidelines) and for adapting existing guidelines to other settings.^3^

Given the advancements and increase in complexity in CPG methods, it is vital that the skills, knowledge and experience of those developing CPGs keep pace.^4^ However, reviews of CPGs in sub-Saharan Africa (SSA) and globally indicate that many of these do not meet standards, for example, they perform poorly on reporting on how they were developed and how editorial independence was maintained.^5,6,7^ Given the substantial potential impact of CPGs, there is a strong rationale for investing in training and capacity building for managing and participating in guideline development and evidence-informed decision-making, comprising aspects linked to convening the CPG group, moving stepwise through a rigorous process and ensuring that resource-efficient methods are used.

Against this backdrop, the Global Evidence, Local Adaptation (GELA) project aimed to maximise the impact of research on poverty-related diseases through enhancing researchers’ and decision makers’ capacity to use research evidence to develop locally relevant CPGs for newborn and child health in Malawi, Nigeria and South Africa (https://africa.cochrane.org/projects/GELA).

The project was implemented over a 3-year period (2022–2025) in Malawi, Nigeria and South Africa, through a multifaceted, multidisciplinary research approach that used primary and secondary research, guideline adaptation methodology, and digital platforms to support dynamic guideline development. Guideline development and adaptation within the project were informed by the GRADE approach, including the use of evidence-to-decision (EtD) frameworks to present evidence within a guideline process, and to facilitate systematic and transparent judgements about the evidence.^8^ This was supported by a capacity building programme, specifically for decision makers such as the guideline development group (GDG) and steering group (SG) committee members (those involved in steering and developing the guidelines), GELA staff members and researchers in the respective countries. Throughout the project, we introduced innovations^9^ in the tools we were using to meet the constraints and needs of the CPG processes in each country, accompanied by a comprehensive evaluation of key aspects of the research, research uptake into policy, and capacity building.

### Aim and objectives

The aim of this article is to evaluate the capacity development of GDG and SG committee members within the GELA project. The objectives are: (1) to describe capacity development activities within GELA; (2) to assess GDG and SG members’ change in knowledge, skills and behaviour related to guideline development; (3) to explore GDG and SG members’ views and experiences of capacity development within GELA; and (4) to reflect on implementation of capacity building activities and share lessons learned.

Evaluation of the capacity development of GELA researchers is addressed elsewhere^10^.

## Research methods and design

### Study design

We used mixed-methods for our summative evaluation, comprising a document analysis, a survey, in-depth interviews and a structured reflection to present a holistic picture of capacity development for the GDG and SG members within GELA.

Our evaluation was informed by the overarching GELA Theory of change^11^ and the Kirkpatrick model^12,13^ for evaluating the impact of training programmes. The GELA Theory of Change depicts the inputs, activities, outputs and outcomes of the GELA project and is detailed in our protocol.^14^ The Kirkpatrick model evaluates training effectiveness across four levels: (1) reaction, assessing participants’ satisfaction with training, (2) learning, assessing change in knowledge before and after the training, (3) behaviour, assessing favourable change in behaviour because of the training and (4) results, assessing the use of new knowledge within the workplace. This model served as our theoretical framework.

### Setting and participants

As part of work package (WP)1 (engage), clinical experts and relevant stakeholders in the field of newborn and child health in Malawi, Nigeria and South Africa were invited to participate in the project as GDG or SG members.^15^ Most of the GDG members had no prior experience with GRADE standard guideline development or the use of the GRADE EtD framework in a structured way for CPGs.^15^ Some of the GDG members had been involved in implementing guideline recommendations or in guideline development that followed processes different from those applied in GELA. Most GDG members were programme managers and health professionals. The SG comprised members representing stakeholders from relevant departments within national health departments in the countries; professional associations; other organisations as suggested by national health departments, such as the ministry of health training and paediatric institutions; and the respective WHO country offices.

### Data collection and analysis

Below, we report on the data collection and analysis per sub-component of our evaluation.

#### Document analysis

We conducted a review of GELA documents to map out the capacity building processes, activities and outputs according to the objectives of each work package within GELA. We identified key project documents per work package and extracted data relevant to capacity development activities, including the types of activities, the aim of these activities and the number of people attending activities and/or sessions per country. To provide an overview of training activities for GDG and SG members, we summarised relevant capacity building initiatives descriptively in a tabular format.

#### Online surveys

We developed a 2-round longitudinal survey following the Kirkpatrick model to assess training impact. At baseline, the survey was divided into four parts: Demographic data, previous training, training needs and confidence in knowledge and skills in guideline development. The follow-up survey was divided into five sections: Demographic data, training attended, training needs, confidence in knowledge and skills in CPG development and behaviour linked to CPG development (see Online Appendix 1). Survey questions were adapted from existing tools.^15,16,17^ For knowledge and skills, we asked participants to rate their confidence in certain skills on a scale from 0 (no confidence) to 100 (completely confident). For behaviour, we asked respondents to rate how often they engaged in certain behaviours on a 4-point Likert-type scale (never, rarely, often, always). Behaviours were related to dealing with conflict of interest, formulating questions, searching for evidence, critically appraising evidence, synthesising and interpreting evidence and applying evidence.

The survey was set up on a secure platform, REDCap (Version 15.0.11). We generated a link to the online survey, which respective country teams sent out to GDG and SG members via email. Some country teams also disseminated the link at formal or informal meetings with GDG and SG members. Participation in the surveys was voluntary, and responses were anonymous. We requested participants to provide a unique identifier when responding to the baseline and mid-term survey so that we could track changes in confidence in knowledge and skills. As we did not include questions linked to behaviour in the baseline survey, we asked respondents to rate behaviour before GELA retrospectively, as well as after GELA in the midterm survey. At baseline, we collected responses from November 2022 to March 2023. Guideline development group members who joined the project later in 2023 were also invited to complete the baseline survey. At follow-up, we collected responses between May 2024 and August 2024, 2 years after the start of GELA.

Data were analysed descriptively. For questions on knowledge and skills, we calculated the mean and standard deviation for each item. For questions on behaviour, we dichotomised data (never or rarely and often or all the time) and presented the number and proportion of participants who indicated that they engage in a certain behaviour often or all the time. Descriptive statistics were generated using R Studio (version 2024.04.1-748, running R version 4.3.2).^18^ At baseline, responses were stratified by countries to explore variations between GDGs. As a result of the low response rate at follow-up, we focused on changes over time among all participants rather than per country. Low response rates and turnover in some of the groups also prevented us from applying inferential statistics as planned in the protocol.^14^ Responses to open-ended questions were summarised.

#### In-depth, semi-structured interviews

We invited 42 GDG and SG members from the three countries via email to participate in semi-structured, individual interviews.^14^ Twenty-seven (64%) participants (16 GDG and 11 SG members) consented and were interviewed online via Zoom or Teams (10 in South Africa, 10 in Nigeria and 7 in Malawi), between November 2024 and January 2025. The average interview time across the countries was 48 min, ranging from 30 min to 1 h 30 min. A member of the research team conducted the interview, and another team member acted as an observer, taking observational notes during the interview. The interviews explored participants’ views and experiences of their general and specific learnings throughout the GELA project. Questions regarding building capacities included a description of the guideline development capacity building meeting they attended, what stood out for them regarding the training activities they participated in, and the challenges they experienced. All interviews were audio-recorded, and where possible, video-recorded, except one participant who preferred not to be recorded. All interviews were transcribed by an outsourced transcriber, reviewed by the team, anonymised, and uploaded by the leader of the analysis team into the Dedoose software package.^19^ We used the framework analysis^20,21^ approach to analyse the data, as outlined in Table 1. The research team met regularly to discuss the analysis, which was also informed by the observational notes.

**TABLE 1:** Steps of the framework analysis of the qualitative data.

Steps	Description of process
Notes and debriefing after interviews	The interviewer and observer in all countries discussed key moments from the interviews. These included the interviewing process and impressions of the participants’ responses. The observer notes and recordings were uploaded to a shared folder.
Transcription, anonymisation and familiarisation of data	We transcribed the recordings using an outsourced contractor. Two members of the research team checked the transcriptions. Two members per country team anonymised and familiarised themselves with the data.
Identification of codes and sample coding	We developed a code list and uploaded it to the Dedoose software package for sample analysis. We held two debriefing sessions and sample coding with nine members across the country teams to finalise the codes prior to analysis.
Coding and summarise codes	At least two members of each country team coded the data and discussed the summary of codes, leading to the development of emerging themes.
Team-based development of themes	The country teams summarised the themes. These were then collated by the lead author and sent to all team members for verification and further inputs.
Revision of themes and interpretation	All nine members revised and finalised the themes and interpretation of these themes with relevant quotations from the participants.

#### Structured reflection

The author team met in-person in February 2025 to reflect on capacity development for GDG and SG members within GELA. We reflected on our experiences with implementing capacity building activities, as well as findings from the survey and interviews. We used Driscoll’s model of reflection^22^ to structure our reflections. This approach is centred around three questions: What? So what? Now what? Firstly, we reflected individually on *what* happened, using Post-it notes to capture key aspects for each of the activities described in Table 2. Secondly, for the *so what*, we discussed individual reflections in the group, focusing on the significance, relevance, as well as similarities and disparities between countries. We noted down key learnings. Thirdly, we applied key learnings to formulate lessons for future practice across activities, as part of the ‘*now what*’. This was an iterative process that involved careful consideration of country-specific experiences and was informed by perspectives of various role-players (GELA capacity development work package leads, facilitators of activities, researchers conducting qualitative interviews) as well as qualitative data collected during the in-depth interviews. Overarching lessons that emerged were refined and are reported as part of our findings.

**TABLE 2:** An overview of capacity development activities for guideline development group and steering group members.^9^

Capacity building activity	Aim	Format	Date offered; number of GDG and SG members attending per country
Primer in systematic reviews (short course)	To increase the capacity to find, read, interpret and consider the use of systematic reviews of effects	8-week online short course Self-study and asynchronous discussion forum Weekly synchronous Q&A session with facilitators Certificate of attendance	March – May 2023: Malawi (*n* = 9) and Nigeria (*n* = 11) August–October 2023: South Africa (*n* = 2), Nigeria (*n* = 2), Malawi (*n* = 2)
Preparing for your participation in a guideline development panel (webinar)	To introduce the guideline development process to the guideline development group panel members	1 h webinar on Zoom Presentation with opportunity for questions and discussions Recording circulated to all GDG and SG members	29 March 2023: Malawi (*n* = 3), Nigeria (*n* = 5) 09 May 2023: Nigeria (*n* = 9), Malawi (*n* = 6)
Guideline Simulation Workshop	To increase capacity and confidence to participate in a guideline development group meeting	Half day workshop In-person Role-play Designated roles sent via email prior to the workshop	26 January 2023: Malawi (*n* = 11) 27 September 2023: South Africa (*n* = 19) 31 October 2023: Nigeria (*n* = 11)
GELA community of practice	To enhance and share knowledge and context-relevant information by supporting the interaction of peers across countries	WhatsApp group for informal discussions Voluntary participation Quarterly online Teams meetings with a 60 – 90 min Structured topics and discussions	28 November 2023: Clinical practice guidelines – what they are and why we need them South Africa (*n* = 7), Nigeria (*n* = 12), Malawi (*n* = 3) 28 February 2024: GRADEing certainty of qualitative evidence South Africa (*n* = 5), Nigeria (*n* = 7), Malawi (*n* = 3) 28 May 2024: Considering health equity in clinical guidelines: Global standards and local application South Africa (*n* = 5), Nigeria (*n* = 11), Malawi (*n* = 4) 04 October 2024: Guideline adaptation: From global to local South Africa (*n* = 5), Nigeria (*n* = 10), Malawi (*n* = 2) 11 March 2025: Using economic evidence in GELA guidelines: Experiences from Malawi, South Africa and Nigeria South Africa (*n* = 1), Nigeria (*n* = 9), Malawi (*n* = 1)

*Source:* Adapted from Kredo T, Durao S, Effa E, et al. Building sustainable capacity to adopt, adapt or develop child health guidelines, Malawi, Nigeria and South Africa. Bull World Health Organ. 2024;102(10):749–756. https://doi.org/10.2471/BLT.24.291564

GELA, Global Evidence, Local Adaptation; GDG, guideline development group; SG, steering group.

### Reflexivity

All authors are part of GELA and had different roles in the project.

We acknowledge that our involvement in the GELA project, and particularly, the activities we are evaluating here, has shaped our own views about GELA. However, through iterative team discussions, we have taken care not to let our own perceptions and experiences influence the quantitative and qualitative findings. The very diverse backgrounds of the team members involved also helped to ensure a diversity of points of view, methodological and theoretical traditions in our approach to data collection, analysis and interpretation. However, our perceptions about the delivery of capacity building activities, in addition to the qualitative data from the interviews, informed our lessons learned. During the reflection, we discussed experiences and validated perceptions through group discussions. We believe that our intricate knowledge about the project has benefited our understanding of the data.

### Ethical considerations

The study was approved by the Human Research Ethics Committee of the South African Medical Research Council (EC015-7/2022), the National Health Research Ethics Committee of Nigeria (NHREC/01/01/2007-22/08/2024) and the College of Medicine Research and Ethics Committee of Malawi (P.07/22/3687).

All participants provided informed consent prior to participating in our study. Respondents to the survey first had to consent to participate (by clicking on a ‘consent’ button) before being able to access the survey questions. This step was built into the online survey. Interviewees had to sign a consent form that was sent to them via email when they accepted to participate in the in-depth semi-structured interviews. All quantitative and qualitative data were anonymised.^14^

## Results

### Capacity building activities within Global Evidence, Local Adaptation

There were four main sets of capacity development activities (Table 2). Most of these were offered online, across GELA partners. The guideline simulation workshop was offered in-person to GDG and SG members in each country. It simulated a real WHO scenario, where participants followed the GRADE EtD process to formulate a dummy recommendation. This served as a practice round for the real EtD process that happened in each country. All GDG and SG members participated in the country-specific guideline development process, from setting priorities to formulating recommendations.

### Impact of capacity building activities on knowledge, skills and behaviour

Between November 2022 and August 2023, 56 GDG and SG members participated in the baseline survey (South Africa, *n* = 18; Nigeria, *n* = 24; Malawi, *n* = 14). At follow-up, 24 months after the start of GELA, 22 GDG and SG members responded (South Africa *n* = 5, Nigeria *n* = 14, Malawi *n* = 3). Of these, 11 participants responded to both surveys (based on self-provided unique identifier), 12/22 (55%) attended the Primer in Systematic Reviews (SR), 4/22 (18%) the webinar on qualitative evidence and 6/22 (27%) attended the guideline simulation workshop. Most participants had postgraduate degrees, and the majority had at least some experience with CPG development. However, few had been involved in systematic reviews (Table 3). Online Appendix 2 provides the detailed survey responses.

**TABLE 3:** Demographics of survey participants.

Variable	Baseline (*N* = 56)			Mid-term (*N* = 22)		
	Mean (SD)	*n*	%	Mean (SD)	*n*	%
**Age (years)**	49 (9)	-	-	49.45 (7.71)	-	-
**Gender**	-	-	-	-	-	-
Male	-	28	50	-	14	64
Female	-	28	50	-	8	36
**Highest academic qualification**	-	-	-	-	-	-
Bachelor or lower	-	4	7	-	3	14
Master’s degree	-	17	30	-	4	18
Professional or Specialty fellowship	-	26	46	-	3	14
Doctoral degree	-	9	16	-	12	55
**Primary role of expertise**	-	-	-	-	-	-
Policy maker or Health ministry staff	-	7	13	-	1	5
Evidence synthesis or guideline methodologist	-	5	9	-	3	14
Health care provider (e.g. Doctor, Nurse, Pharmacist, Allied health practitioner, Traditional practitioner)	-	26	46	-	17	77
Healthcare or Programme manager	-	11	20	-	0	0
Other	-	7	13	-	1	5
**Experience with systematic reviews**	-	14	25	-	8	36
**Presently involved in a review in progress**	-	4	7	-	1	5
**Experience with clinical guideline development**	-	36	64	-	16	73

SD, standard deviation.

Respondents to the midterm survey reported higher levels of confidence in skills related to CPG development compared to those who responded at baseline (Table 4). The most notable differences in self-perceived confidence (20-point increases) were seen for skills related to appraising and interpreting systematic reviews of effects, including concepts related to GRADE certainty of evidence and summary of findings tables. Furthermore, respondents at midterm also reported higher confidence in participating in the guideline panel process.

**TABLE 4:** Participants’ confidence in their skills related to clinical practice guideline development, by survey round (0 = no confidence to 100 = completely confident).

Skills related to CPG development	Baseline assessment (*N* = 56)		Midterm assessment (*N* = 22)	
	Mean score	SD	Mean score	SD
Explain general concepts of Conflicts of Interest	68	24.20	71	22.36
Explain the importance of multistakeholder input	71	24.19	70	22.67
Participate in guideline panel process	55	24.07	72	22.01
Generate a structured question for a quantitative evidence synthesis	57	25.85	69	23.95
Explain the different types of evidence syntheses	54	27.35	67	22.70
Appraise existing Systematic Reviews or Evidence Syntheses	49	27.95	70	25.25
Interpret a forest plot	49	30.95	71	25.98
Describe the concept of GRADE and evidence certainty	50	28.36	70	26.22
Describe the elements of an evidence profile or summary of findings table	45	29.66	68	26.74
Interpret an evidence profile or summary of findings table	46	30.20	66	25.99
Describe the importance of values and preferences in making recommendations	53	28.08	67	24.84
Describe the importance of resource use in making recommendations	59	23.24	72	25.85
Describe the importance of equity, acceptability and feasibility to making recommendations	57	23.85	70	25.88
Describe the importance of appropriate wording of recommendations and identify elements required for clear and actionable recommendations	61	22.19	71	24.44
Describe the importance of updating guidelines	67	25.12	76	26.13
Develop a structured question for a Qualitative Evidence Synthesis	44	24.79	61	23.99
Find qualitative research evidence	52	25.55	59	24.19
Interpret qualitative research evidence	51	23.46	57	26.01
Use an evidence-based adaptation framework to adapt a guideline	45	25.59	55	27.84

SD, standard deviation; CPG, clinical practice guideline; GRADE, Grading of Recommendations, Assessment, Development, and Evaluation.

At midterm, we asked participants to indicate how often they adopted key behaviours related to CPG development, further to their participation in GELA (as compared to before, assessed retrospectively) (See Table 5). Results show that they engaged more frequently in all the behaviours listed below after having participated in GELA activities. Fewer respondents reported that they often or always engaged in behaviours linked to the synthesis and interpretation of evidence, compared to other behaviours (Table 5).

**TABLE 5:** Comparison of participants’ behaviours linked to clinical guideline development over time (assessed at midterm) (*N* = 22).

Behaviours related to guideline development	Before GELA (retrospective assessment)		After GELA	
	*n*	%	*n*	%
**Conflict of interest**				
I often or always declare my conflicts or interests when participating in guideline development.	12	54	21	96
**Formulating questions**				
I often or always question healthcare practices for the purpose of improving the quality of care/service delivery.	13	59	20	91
I often or always participate in the formulation of clinical and/or public health practice guideline questions.	4	18	16	73
**Searching for evidence**				
I often or always search for research evidence (including guidelines) to answer healthcare questions.	11	50	21	96
**Critical appraisal of evidence**				
I often or always participate in the critical appraisal of individual research studies to determine their strength and applicability to healthcare practice.	7	32	17	77
I often or always participate in the critical appraisal of synthesised evidence (such as clinical practice guidelines, evidence-based policies and procedures, and evidence syntheses).	5	23	14	64
**Synthesis and interpretation of evidence**				
I often or always participate in the synthesis and interpretation of effectiveness evidence gathered to formulate recommendations for healthcare practice.	6	27	12	55
I often or always participate in the synthesis and interpretation of qualitative evidence gathered to formulate recommendations for healthcare practice.	2	9	13	59
I often or always participate in the synthesis and interpretation of economic evidence gathered to formulate recommendations for healthcare practice.	3	14	10	46
**Application of evidence**				
I often or always integrate evidence gathered from healthcare expertise, community and/or patient preferences, and local context with research evidence to formulate recommendations for healthcare practice.	5	23	13	59
I often or always acknowledge implementation considerations related to recommendations for healthcare practice.	8	36	20	91

GELA, Global Evidence, Local Adaptation.

### Guideline development group and steering group members’ views and experiences of capacity development

Fourteen males and 13 females participated in the interviews. Most of the interviewees (*n* = 22) had high-level qualifications (Master’s degree and above) and had professional skills in health fields, with a mix of academics, clinicians such as paediatricians and neonatologists, and policymakers. More information regarding participants’ demographics can be found in Online Appendix 3. Below, we present a summary of emerging themes. Supporting quotes for each theme can be found in Table 6.

**TABLE 6:** Participants’ quotes supporting Theme 1 to Theme 7.

Theme	Participant quotes
Theme 1: Enhanced understanding and capacity development through a rigorous guideline development process	‘I didn’t have a lot of formalised understanding of like how to GRADE evidence and the quality of evidence and that … that was a big thing that I did get out of the GELA project. I mean understanding how to qualitatively actually appraise the evidence, I mean that was not something that we normally do, so that was a huge learning for me.’ (Country A, GDG, Participant 5) ‘Yes, however I still note that there is much difference in this GELA project … we have been oriented on what to do during the simulation project. I was exposed to both local and international experts. I learned a lot.’ (Country B, GDG, Participant 4) ‘That economic evidence that you actually presented because you know, in our setting here with low- and middle-income countries and when we talk about costs it’s very, very important … So I think that aspect actually interested me, the economic aspect of the evidence we used. And we saw that actually it was cost effectiveness. I don’t know whether I will say it was feasible, let me say it’s actually feasible in our setting.’ (Country C, GDG, Participant 4) ‘So I think it has been very enlightening for me as a person, both the capacity development and trainings. The primers in systematic review was a big one for me and then of course the real meetings of how to really develop recommendations for guidelines was really, really very interesting for me. So I think those are the major ones.’ (Country C, GDG, Participant 3) ‘Yes, yes I really in my background have done very little or none of the qualitative studies. So it took a bit to wrap my head around it, but once you are sitting with people in a room who are experts at that you are able to learn from them. And hence the courage now to actually take on a master’s student who wants to do this qualitative study.’ (Country A, GDG, Participant 2)
Theme 2: Intensive nature of some capacity development activities	‘That particular training was quite intense, very interesting and I was happy I participated in that because it opened my eyes to the importance of systematic reviews and how to interpret them and the significance of it in making guidelines and all. So I feel that that training is important for most people who will be involved with developing guidelines and all, handling systematic reviews. It was an eye opener for me … So apart from the fact that it was intense, I feel that people should take it really as if you are going for a course and a certificate course kind of.’ (Country C, GDG, Participant 3) ‘It was an excellent course [*Primer in Systematic Reviews*], a lot of knowledge gained there. I am thinking now about how to actually practically do systematic reviews, so I was inspired by that.’ (Country B, SG, Participant 1)
Theme 3: Strengthening collaboration and creating opportunities for future networking	‘I think they did enhance our connection and collaboration, and I think you get to interact more with other practitioners and other scientists over and beyond the activity that you are doing and so I think was quite a good one.’ (Country B, GDG, Participant 2) ‘I think what I learned from some of our meetings, every voice is important, every opinion should be considered on its own merits, irrespective of who is giving the opinion. So, I think it has helped me to see the need for collaboration, working with others and realising that the more opinions we have the more we bring to the table, the better the recommendation will be based on the evidence that we have. So, I think that beyond just the GDG it has influenced my interaction with others at various levels.’ (Country C, GDG, Participant 3) ‘The facilitation skills were great in that as I said even though there were many of us and very highly opinionated people by the way, we were able to – you don’t know neonatologists, we are prima donnas … We were able to really come to a conclusion and then begin to work seriously on the one topic we said we were going to do. So just watching how they were able to facilitate these things seamlessly and make all of you feel that you are part of that decision and owning that decision at the end for me was good.’ (Country A, GDG, Participant 2)
Theme 4: Valuing a more thorough and rigorous approach to guideline development	‘I would have liked to have been an integral part of using the GRADE approach because I had obviously read about it and the evidence that we see we know about the GRADE, but I haven’t done that sort of study myself. So, as a skill that would have been good for me but as I said it was just not possible in the last year to find the space and time to do that. But I thought it would be a two-way learning that the project would extract information from me from my experience but in return I would gain the skill of being able to do a rigorous evidence-based guideline approach. And unfortunately, I didn’t get that out because of my own time constraints.’ (Country A, GDG, Participant 3) ‘… With this GELA project it means I would have benefited a lot because this project is also about ensuring that the guidelines that are being developed are evidence-based, so I would have benefited a lot. And this would also have helped me in my day-to-day practice, like I say that in [our department] you cannot do without – you cannot manage clients, you cannot manage patients without checking what is the evidence all about, yes so I could have benefited a lot.’ (Country B, GDG, Participant 4)
Theme 5: Challenges: Limited internet connectivity, time constraints and unhelpful sessions	‘… [*T*]he issues I had with all those things was where I was the internet was my main limitation, so effective participation, so it was on and off. The main points were heard.’ (Country C, SG, Participant 2) ‘… [*S*]o online, the convenience aspect in terms of you can do it from anywhere you are, but the challenge is that you are in the office, the disturbances can be there. It’s different from – like for example the simulation one was onsite and physical and you know that if you are there you are there, you are just doing that and it was quite an effective way and possibly that’s why they chose that at least the simulation should be physical and it was quite a good choice.’ (Country B, GDG, Participant 2) ‘I was a bit engaged to attend to most of the trainings, but still I hope for those people who have attended have benefited a lot …’ (Country, B, GDG, Participant 4) ‘Yeah, I can remember going to the last one that was presented with the local adaptation one, but that one I found was a bit geared towards, I think students that’s doing a course. So, they were more giving feedback. They were not giving any new information. It was just a like a feedback session. So I didn’t find a lot of value in that one.’ (Country A, GDG, Participant 4) ‘[*I*]t was interesting that we were invited as a national brains trust treasure because of our subject expertise. But then subsequently we were – we were treated like – like junior faculty who could really benefit from a little bit of – of career development and advancement.’ (Country A, GDG, Participant 1)
Theme 6: Unmet expectations in involvement in gathering and appraising evidence	‘I thought about how it [*the guideline process*] could have been done differently … I would want to have the list of the included evidence … it was interesting that we were invited as a national brains trust treasure because of our subject expertise. But then subsequently we were – we were treated like – like junior faculty who could really benefit from a little bit of – of career development and advancement.’ (Country A, GDG, Participant 1) ‘I think maybe getting involved with the actual guideline writing would have been great and not just the recommendations. I know, I think there was a theoretical – if I recall well, I hope I am not confusing it with something else, systematic reviews, I hope it was part of the GELA if I recall very well.’ (Country B, GDG, participant 2) ‘So my expectations, at first I thought I will be involved much … thought that I will be involved in generating … and grading them whether they – based on the scientific evidence and then summarising everything related to the nutritional issues – that was my first expectations. But however in the process I learned that my core role was just to make some recommendations using evidence-to-decision-making.’ (Country B, GDG, participant 4)
Theme 7: Sustaining training and institutionalising guideline processes	‘From the point of view of the skills training I think it’s critical, because in Africa we also want to be comparable to places in high income countries, so that we have similar sets of skills available.’ (Country A, GDG, Participant 4) ‘So basically I think we really need to therefore institutionalise this GELA guideline development processes], so it should not come as a project as it is, but it has to come as a – to be mainstreamed in all areas.’ (Country B, SG, Participant 3) ‘So having something like GELA really helps to bring and institutionalise that within the government system. And such that it could actually give us an opportunity to also consult with the private sector … So this is what I see it being beneficial going forward and we see how government can easily be overwhelmed with such processes or having some support as this would be great.’ (Country A, SG, Participant 4)

GDG, guideline development group; GELA, Global Evidence, Local Adaptation; GRADE, Grading of Recommendations, Assessment, Development, and Evaluation; SG, steering group.

#### Theme 1: Enhanced understanding and capacity development through a rigorous guideline development process

Some of the GDG members who were involved in the guideline development meetings and/or training activities, such as the webinars and the Primer in SR short course, reported an enhanced understanding of how to ensure rigour in the CPG development process. They also reported that their capacity to be involved in such a process in the future had been enhanced. Other GDG members who were part of the guideline simulation workshop reported learning because of their exposure to other experts within the GDG group. Many participants had previously been involved in guideline development and identified differences in their understanding because of the GELA project. For example, several participants gave examples of using newly gained knowledge and skills, in particular the use and value of qualitative and economic evidence in guideline development.

#### Theme 2: Intensive nature of some capacity development activities

Some of the participants mentioned that they benefited from the capacity development activities, such as the Primer in SR short course. However, they also shared that this course’s content was intense and they had to cover a lot of topics in a short period of time. Nonetheless, they suggested others working in the field should also be offered this opportunity. Other participants reported that, after attending the Primer in SR short course, they were planning to conduct systematic reviews, suggesting that they felt spurred to move beyond *using* reviews to *doing* reviews.

#### Theme 3: Strengthening collaboration and creating opportunities for future networking

Many participants reported that GELA capacity development activities strengthened collaboration with colleagues in similar research areas. This included the shared knowledge gained in activities such as the guideline simulation workshop or community of practice (Table 2), and the potential for future networking.

#### Theme 4: Valuing a more thorough and rigorous approach to guideline development

Several participants who had been involved previously in guideline development mentioned that ‘expert opinion’ was often the only evidence used in these guidelines. They therefore valued the more thorough and rigorous approach in collecting and appraising evidence that was adopted in GELA.

A few of the participants who could not attend some capacity development activities reported that attendance would have been beneficial in terms of building their capacity to engage in a rigorous guideline development process in GELA and beyond. These participants came mainly from South Africa and had more extensive guideline development experience than participants from Malawi and Nigeria.

#### Theme 5: Challenges: Limited internet connectivity, time constraints and unhelpful sessions

A few participants had limitations in effective engagement in the capacity development activities because of the online nature of some of the courses, which required stronger internet connectivity. Others highlighted time constraints to attend some of the sessions, while others did not find value in the way some of the sessions were structured.

#### Theme 6: Unmet expectations in involvement in gathering and appraising evidence

Some participants reported that their expectations were not met because they envisaged being more involved in the process of gathering and appraising the evidence that was presented at the GDG panel meeting. For others, it was initially unclear what their roles were.

#### Theme 7: Sustaining training and institutionalising guideline processes

Some of the participants indicated the perceived disparities in the skills to engage in CPG development activities in the Global South compared to the Global North. Participants suggested ways to provide more training as well as sustain the skills gained from the GELA project. Some suggested that governments should play central roles in coordinating similar projects and/or institutionalising this guideline development processes. The aspect of institutionalisation came up strongly among the participants, and they suggested different ways this could be done. Some suggested it should be mainstreamed into every government sector. Others thought the private sector must be involved to assist governments to implement such projects effectively.

### Lessons learned

The following overarching lessons emerged from our structured reflections as GELA researchers:

**Consider diversity and differing priorities among learners:** Across GELA, learners were diverse with regard to professional and clinical background, experience in guideline development, knowledge about evidence-informed decision-making and evidence synthesis, and cultural background. This made it difficult to ensure that training activities were relevant and met all the needs and expectations of GDG and SG members across the three countries. Future endeavours should consider how different teaching methods can be used to strengthen capacity among learners with varied backgrounds and competencies, and elicit feedback from participants on the usefulness of these approaches. Furthermore, broadening capacity building options could meet different needs and priorities.

**Consider ways of clarifying expectations and learning needs at the beginning of the project:** This includes GDG members’ expectations about their involvement in the project and opportunities for capacity development – both in terms of content and format. Some GDG members expected to be part of the team producing the evidence, some did not like the online format and thus did not participate, while others expected to be compensated for their time. Although each GDG member received a Terms of Reference when they joined GELA, there still seemed to be uncertainty about these issues, specifically among participants who joined the project at a later stage. Future projects should ensure that expectations are clearly communicated and reiterated if necessary, and learning needs are addressed as far as possible.

**Consider choosing country-specific champions to drive a community of practice:** A community of practice should not only be content-driven, but equally needs to focus on networking, collaborating and sharing of interests and resources.^23,24^ While interaction during online community of practice meetings was good and feedback was positive, engagement outside of the formal meetings was lacking. Future projects should consider appointing country-specific champions to encourage informal conversations and networking to increase ownership, interaction and sustainability of a community of practice.

**Consider using a framework to anchor capacity building activities:** Global Evidence, Local Adaptation offered various activities to GDG and SG members (Table 2), aiming to increase the use of evidence in decision-making. However, the bigger picture in terms of how these activities fit together and how they fed into the guideline development process was not always clear. In future, it could be useful to structure learning according to the GRADE EtD framework in an explicit way, to help learners understand the overall goal of capacity development, as well as the value of each activity and its relationship to the guideline development process.

**Consider scaffolding learning as far as possible:** Planned activities should build on each other, scaffolding the learning opportunities longitudinally. Towards this, it would be useful to start with the building blocks of evidence-informed decision-making (e.g. Primer in SR short course) before learners experience the guideline and EtD process through the guideline simulation workshop and other activities. This would allow a sound baseline of using and interpreting systematic reviews, especially for those with limited experience in evidence synthesis, before attending GDG training or participating in a real guideline panel meeting. Furthermore, GELA had capacity building goals as wells as goals related to guideline development, and these did not entirely mesh in terms of timelines and emphasis. It may be helpful to provide recordings of missed training activities to members who join late to close the learning gap prior to attendance at GDG meetings.

**Consider including authentic and experiential learning:** The guideline simulation workshop provided GDG members with the opportunity to experience a guideline meeting in a safe and real-world setting before participating in the actual GDG meetings in their respective countries. This enabled learners to connect didactic knowledge with practical application of skills needed to participate in a CPG process. Across countries, this workshop stood out as being invaluable, highlighting the value of simulation-based and experiential learning approaches. To maximise learning, future projects should include various approaches that foster learner-centred experiences and real-world connections.

**Consider integrating capacity building and learning by doing:** Although planned training activities played a key role in increasing knowledge, skills and behaviour, it is likely that capacity was also built because GDG and SG members participated in the project itself. To scale up capacity building, future endeavours should consider integrating planned training activities and learning by doing. This could support the process towards institutionalising rigorous and transparent guideline development in national systems.

## Discussion

This article aimed to evaluate capacity development of GDG and SG committee members GELA. Capacity building activities included formal short courses, webinars, in-person guideline simulation workshops and an online community of practice. Our needs assessment (baseline survey) at the beginning of the project highlighted learning needs across all evidence synthesis and guideline development domains. Table 7 provides a summary of our findings according to the four levels of Kirkpatrick’s model to evaluate training.

**TABLE 7:** A summary of findings according to Kirkpatrick’s model to evaluate training.

Kirkpatrick level	Survey Baseline (before GELA) *N* = 56, Midterm (after GELA) *N* = 22	Interviews (*N* = 27)
Reaction	Not assessed	**Theme 2:** Intensive nature of some capacity development activities **Theme 4:** Valuing a more thorough and rigorous approach to guideline development **Theme 5:** Challenges: Limited internet connectivity, time constraints and unhelpful sessions
Learning	More than 20% points increase in the mean score from baseline to follow-up for the following skills: Appraise and interpret systematic reviews of effects Interpret forest plots Describe GRADE certainty of evidence Describe and interpret evidence profiles	**Theme 1:** Enhanced understanding and capacity development through a rigorous guideline development process
Behaviour	10 or more additional respondents indicating that they often or always engage in the following behaviour after GELA: Formulating questions Searching for evidence Critical appraisal of evidence Acknowledging implementation considerations	**Theme 6:** Unmet expectations in involvement in gathering and appraising evidence
Results	Not assessed	**Theme 3**: Strengthening collaboration and creating opportunities for future networking **Theme 7:** Sustaining training and institutionalising guideline processes

GELA, Global Evidence, Local Adaptation; GRADE, Grading of Recommendations, Assessment, Development, and Evaluation.

The survey found that participants’ confidence in skills required for CPG development increased, and their self-reported behaviours linked to CPG development changed through GELA activities. However, it is likely that the change in knowledge, skills and behaviour over the course of GELA was not only because of capacity development activities but also as a result of learning by doing, as many respondents indicated that they did not attend courses but participated in the GELA guideline development process in their respective countries. Therefore, apart from the planned activities within GELA,^14^ capacity development also occurred implicitly during the ‘actual’ priority setting and guideline development meetings, and associated team discussions. Here, they received hands-on experience in priority setting, using evidence and participating in the guideline development process. For some, this would have provided them with opportunities to apply and potentially extend the knowledge and skills they had obtained from the formal capacity building activities in real-world settings.

The interviews complemented the key survey findings, which subsequently shaped the team’s reflections on the importance of tailoring activities that are underpinned by appropriate educational pedagogy, such as adult learning principles and experiential learning theory.^25,26^ For example, we used simulation as a teaching event, mimicking a guideline development panel meeting with elements of role play. The simulation provided an authentic experiential opportunity, allowing participants to put into practice various evidence-informed decision-making competencies, including a practical application of GRADE for guidelines^8^ in making a recommendation, and an appreciation for both qualitative and quantitative evidence. A similar guideline simulation workshop has been developed and implemented in Norway by the Norwegian project partners, and a recent evaluation thereof highlights the potential value in increasing active participation in a real guideline development process.^27^ In GELA, we found that teaching and learning activities, such as the simulation, contributed to the increased confidence of participants in guideline development processes and decision-making spaces (especially those new to the process). Indeed, other studies have also emphasised the value of authentic learning environments for CPG training. One study reported on guideline development training of European Neurology Residents and found that exercises mimicking the guideline development process were effective to enhance understanding and raise awareness of guideline development.^28^ Another study from the USA, reporting on CPG training for patient representatives, also found that experiential learning and roleplay were key in learning about the guideline development process.^29^

Furthermore, self-reported behaviour changes were seen in the guideline development activities, such as the formulation of clinical and/or public health practice guideline questions, searching for research evidence to answer healthcare questions and participating in the critical appraisal of individual research studies to determine their validity and applicability to healthcare practice. Many participants were also positive about how the process enhanced their collaboration with in-country colleagues in similar research interests. Drawing on the views and experiences of GDG and SG committee members regarding the positive impact of GELA activities, it was not surprising to see that many suggested ways to provide more training, as well as sustain the skills gained from the GELA project and/or institutionalise these guidelines development processes.

Indeed, capacity development is likely a cross-cutting and necessary principle in guideline development, not an optional extra. Without sound training in evidence-informed decision-making and guideline processes, or if those making the decisions (such as clinicians and policymakers) lack this training, we undermine the credibility of recommendations and trust in the outputs, and risk harmful recommendations.^30^ The ongoing shifts and evolution in guideline development methods often place considerable pressure on guideline panels and steering committees, such as seen in living and rapid guideline methods.^31,32,33^ Thus, as the complexity of guideline development increases, we must sufficiently equip those involved. This includes emphasising exposing GDGs to different streams of evidence, such as qualitative and economic evidence, beyond the usual focus on effectiveness evidence and interpretation thereof. A recent survey conducted among guideline developers in the Netherlands also highlighted the importance of training for CPG development and incorporating aspects linked to economic and qualitative evidence.^34^

Our findings are based on a document analysis, an online survey, individual, in-depth interviews and a structured reflection. We thus provide a holistic picture of our experience with strengthening capacity in CPG development of GDG and SG members from South Africa, Malawi and Nigeria. As far as we know, this is the first study to report on CPG capacity development for GDG and SG members in SSA. All authors were involved in the implementation of GELA, and were thus able to critically reflect on capacity development activities and understand the context within which these were implemented. These reflections, together with findings from in-depth interviews, led to rich discussions on lessons learned. However, we are aware that our involvement with GELA has the potential to shape our interpretation of data.

Although we invited all GDG and SG members to attend various capacity development activities, participation varied across countries, with good representation from Nigeria, and less so from South Africa and Malawi. Similarly, most respondents to both surveys were from Nigeria. Presumably, the higher participation by Nigerian participants was because of ongoing involvement as members of guideline committees of professional groups and the desire to upskill for subsequent independence in those positions. It may also be related to the use of different reminder approaches to encourage participation. Furthermore, GDG and SG members in Malawi preferred in-person rather than online training, which probably also impacted participation in activities. We therefore acknowledge that the findings may not be a good representation of the full range of views of participants from South Africa and Malawi.

Overall, the response rate to the surveys, particularly the mid-term survey, was low despite numerous reminder emails. We did not collect information on reasons for not responding to the survey. Guideline development group and SG members were busy clinicians, who participated in the GELA project in addition to their full-time jobs, and were not compensated for their time. It is also possible that there were too many requests over the course of the project, and initial enthusiasm might have decreased by the time of the evaluation. Lastly, respondents might have been hesitant to respond honestly and therefore preferred not to respond. We acknowledge that the sample of respondents (specifically to the mid-term survey) may have biased the results. In the mid-term survey, most respondents indicated that they did not participate in the capacity development activities.

Our survey tool was adapted from two existing tools.^14^ We used the competencies (for novices and advanced beginners) proposed in the work by Sultan et al.^15^ as a basis for questions on confidence in guideline development knowledge and skills in the survey, but not to develop learning activities. Even though there might have been a slight disconnect between what participants learned and the questions in the survey, we are confident that we covered most competencies through the various activities and participation in GELA.

Although we asked GDG and SG members to rate their confidence in their knowledge and skills at the beginning of the project, we did not include a question on behaviour in the baseline survey. Instead, we asked respondents of the mid-term survey to rate their behaviour before GELA retrospectively. Recollection bias may have led to an inaccurate assessment of behaviour frequency at baseline.

Future research should build on our experience to explore how capacity in evidence-informed decision-making and CPG development can be strengthened in a sustainable way and integrated into routine training. This includes exploring barriers to engaging in capacity development activities, learner preferences in terms of the format of activities, and the role of mentorship between experienced and novice GDG members. Training should be based on learners’ needs and baseline skills. In our sample, self-perceived confidence in finding and interpreting qualitative research evidence, as well as methods to adapt guidelines remained low.

Similarly, behaviour linked to synthesising and interpreting effectiveness, qualitative and economic evidence for guideline development remained low. While GDG members wouldn’t be expected to carry out all these tasks themselves, they need a good understanding of how each of these steps is performed to make informed decisions based on the evidence presented to them. Further learning opportunities should address these needs. Further exploration of simulation-based learning (such as guideline panel simulations) and its impact on participants’ confidence and competence, not only for guideline panellists but also for those facilitating the process, should be further explored. Simulation could thus act as a valuable ‘play-ground’ for both prospective panellists and methodologists to hone their skills for the real-world setting, however, research on the impact of simulation for guideline panels remains sparse. Lastly, there is a need to explore how guideline processes can be institutionalised within national borders and across, where knowledge, process and systems sharing is paramount.

## Conclusion

The GELA project was an important opportunity for GDG and SG members in South Africa, Malawi and Nigeria to learn about and participate in a rigorous guideline development process. The multifaceted approach comprising formal courses, experiential learning and networking was seen by participants to facilitate capacity development in evidence-informed decision-making. The sustainability of training initiatives and the institutionalisation of rigorous guideline development processes remain challenging in the study settings, as these require time and resources, including experienced guideline methodologists. Continuing to support networking and collaboration between evidence producers, evidence users and guideline methodologists is important, including through both formal and informal initiatives.
